# Endogenous Ketone Bodies Are Associated with Metabolic Vulnerability and Disability in Multiple Sclerosis

**DOI:** 10.3390/nu17040640

**Published:** 2025-02-11

**Authors:** Taylor R. Wicks, Irina Shalaurova, Anna Wolska, Richard W. Browne, Bianca Weinstock-Guttman, Robert Zivadinov, Alan T. Remaley, James D. Otvos, Murali Ramanathan

**Affiliations:** 1 Department of Pharmaceutical Sciences, University at Buffalo, The State University of New York, Buffalo, NY 14214, USA; trwicks@buffalo.edu; 2 LabCorp Diagnostics, Morrisville, NC 27560, USA; 3 Lipoprotein Metabolism Laboratory, National Heart, Lung, and Blood Institute, National Institutes of Health, Bethesda, MD 20892, USA; 4 Biotechnical and Clinical Laboratory Sciences, University at Buffalo, The State University of New York, Buffalo, NY 14214, USA; 5 Department of Neurology, University at Buffalo, The State University of New York, Buffalo, NY 14203, USA; 6 Buffalo Neuroimaging Analysis Center, University at Buffalo, The State University of New York, Buffalo, NY 14203, USA; 7 Center for Biomedical Imaging at Clinical Translational Science Institute, University at Buffalo, The State University of New York, Buffalo, NY 14203, USA

**Keywords:** ketone bodies, beta-hydroxybutyrate, acetoacetate, lipid peroxidation, antioxidant defense, metabolomics, multiple sclerosis

## Abstract

**Purpose**: Ketone bodies could be useful biomarkers in multiple sclerosis (MS) because the pathophysiological processes underlying MS disease progression induce metabolic stress. The purpose was to assess the relationships of ketone bodies with biomarkers of metabolic, inflammatory, and oxidative stress in MS. **Methods**: Blood samples and neurological assessments were obtained from 153 healthy controls (HC), 187 relapsing-remitting (RRMS), and 91 progressive MS (PMS) patients. AcAc, BHB, and acetone were measured using proton nuclear magnetic resonance spectroscopy. Indices of inflammatory vulnerability (IVX), metabolic malnutrition (MMX), and metabolic vulnerability (MVX) were computed from the NMR profiles. Cholesterol, apolipoprotein, lipid peroxidation, and antioxidant profiles were obtained. Regression analysis adjusted for age, sex, body mass index, and HC, RRMS, or PMS disease status. **Results**: AcAc and BHB levels were greater in MS compared to HC. BHB and ketone bodies were positively associated with disability on the MS Severity Scale and ambulation time. BHB was positively associated with IVX, MMX, and MVX. AcAc was positively associated with MMX and negatively associated with IVX and MVX. Total ketone body concentration was positively associated with MMX and MVX. BHB and AcAc levels were negatively associated with the amino acids alanine, valine, and leucine. **Conclusions**: Ketone bodies are associated with inflammatory vulnerability, metabolic vulnerability, and ambulatory disability measures in MS.

## 1. Introduction

Multiple sclerosis (MS) is a neurodegenerative disease of the central nervous system (CNS) causing progressive physical and cognitive disability. MS diagnosis is based on magnetic resonance imaging biomarkers of blood-brain barrier injury, inflammatory lesions, and serum or cerebrospinal fluid biomarkers of inflammation and neuroaxonal injury [1].

**Figure 1:**
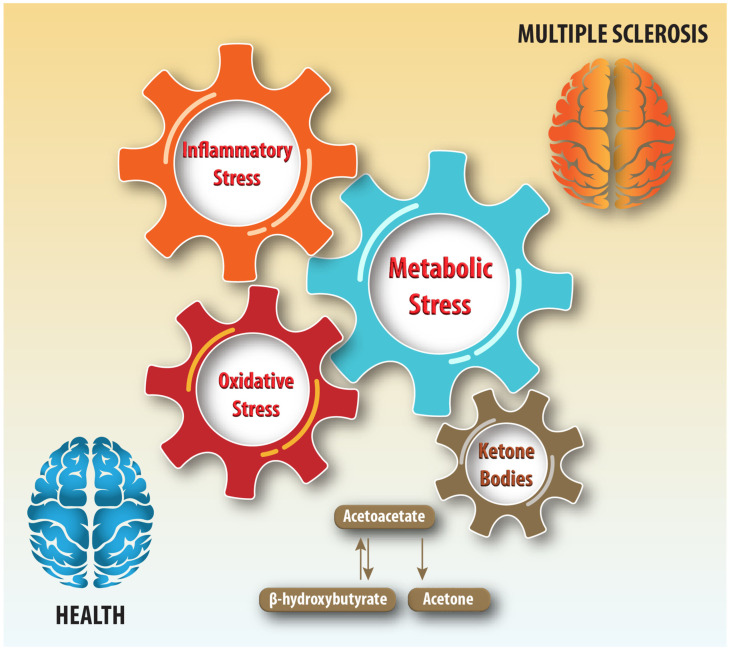
Schematic illustration of ketone bodies and inter-dependencies of inflammatory, metabolic, and oxidative stress in health and multiple sclerosis.

Metabolic dysfunction resulting from impaired mitochondrial function and carbohydrate, lipid, and protein metabolism alterations can lead to central nervous system (CNS) injury [2]. Metabolic vulnerability can impair remyelination and repair, which require structural building blocks and energy. This research focuses on ketone bodies, which are markers of metabolic stress and important substrates for meeting the energy needs of the brain when glucose levels are low [3]. Ketone bodies could be useful biomarkers because the inflammatory, oxidative stress disturbances, and neurodegenerative pathophysiological processes underlying MS disease progression induce metabolic stress (Figure 1).

Ketone bodies consist of acetoacetate (AcAc), β-hydroxybutyrate (BHB, 3-hydroxybutyrate), and acetone, which are produced by ketogenesis from acetyl coenzyme A (acetyl-CoA) in mitochondria. Ketogenesis occurs constitutively but is activated and becomes an important energy source during glucose insufficiency. Ketogenesis occurs primarily in the liver, but astrocytes are also capable of ketogenesis [4,5]. Ketogenesis is activated when acetyl-CoA accumulates due to increased β-oxidation of free fatty acids and is converted to AcAc in a series of enzyme-catalyzed reactions [3]. Most of the AcAc is then reduced to BHB by the BHB dehydrogenase enzyme. Non-enzymatic and enzymatic decarboxylation of AcAc yields acetone [3].

During ketolysis, energy is produced from ketone bodies by converting AcAc back to acetyl-CoA, which enters the citric acid cycle to generate adenosine triphosphate (ATP). The liver is a net exporter of ketone bodies because it lacks succinyl-CoA-3-oxaloacid CoA transferase (SCOT). Because SCOT is an enzyme essential for ketolysis, the liver cannot utilize the ketone bodies it produces. Circulating ketone bodies are transported into the brain by monocarboxylate transporters, SLC16A1 (MCT1) and SLC16A7 (MCT2) [6,7]. During periods of glucose insufficiency, the brain relies on ketolysis for its energy needs because brain mitochondria are inefficient at utilizing fatty acids via β-oxidation [6,8].

While ketone bodies are an alternative energy source for the brain, they could have double-edged consequences in MS. Pathologically, high ketone body levels that can occur in diabetes, alcohol use disorder, and starvation can cause life-threatening ketoacidosis. However, ketolysis has been associated with enhanced reactive oxygen species (ROS) and increased histone acetylation [9]. Neurons and oligodendrocytes are susceptible to damage from ROS due to their reliance on mitochondrial respiration and energy [10]. Increased ROS also leads to M1 proinflammatory microglial activation, inducing the production of pro-inflammatory cytokines such as tumor necrosis factor-α (TNFα), interleukins (IL)-1β, and IL-6, as well as the chemokine CCL2 [9].

However, much of the current understanding of the roles of ketone bodies in brain metabolism and neurodegeneration is derived from pharmacological models and clinical studies of nutritional ketosis and diabetic ketoacidosis, which represent severely perturbed metabolic homeostasis. The relevance of these findings to understanding the effects of typical ketone body concentrations in MS is uncertain. Chronic upregulation of ketone bodies can produce metabolic dysregulation, leading to the activation of further pathophysiological processes involved in MS. AcAc, BHB, and acetone have been found to be increased in MS compared to healthy controls, contributing to altered energy metabolism [11].

The objectives of this research were to investigate the associations of ketone bodies with composite metabolic dysfunction metrics, oxidative stress, and antioxidant defense biomarkers, and with neurodegeneration and neuroaxonal damage in people with MS.

## 2. Methods

### 2.1. Study Design

**Study Setting and Design:** This cross-sectional study was conducted at the Jacobs Multiple Sclerosis Center for Treatment and Research of the University at Buffalo in Buffalo, NY.

**Informed Consent:** The University at Buffalo Human Subjects Institutional Review Board approved the study protocol (MODCR00009015), and written consent was obtained from all patients in line with the Declaration of Helsinki.

**Clinical Assessment:** Neurological assessments were performed, and demographic information was collected.

Healthy Control (HC) participants were required to have a normal physical and neurological examination.

The exclusion criteria for participants with MS were the use of corticosteroids, relapse in the preceding 30 days before the study, and pregnant or nursing mothers. The inclusion criteria for the sub-study were the availability of one or more NMR-derived biomarkers and ≥18 years old.

Diagnosis of MS disease course was made by a licensed neurologist based upon the 2010-revision of the McDonald Criteria. Expanded Disability Status Scale (EDSS) was utilized to measure disability. Multiple Sclerosis Severity Scale (MSSS) scores were calculated from the EDSS scores and disease duration. Ambulation time was obtained from recorded 25-foot walk.

**Serum Biomarker Analysis:** Non-fasting blood samples from participants were processed within 24 h to separate the serum and plasma and frozen in aliquots at −80 °C.

### 2.2. Serum Nuclear Magnetic Resonance (NMR) Analysis

Serum samples were analyzed with proton NMR by LabCorp (Research Triangle Park, NC, USA). The NMR spectra were analyzed with the LP4 algorithm to compute concentrations for the ketone bodies BHB, AcAc, and acetone, the amino acids alanine, valine, leucine, and isoleucine, citrate, and small high-density lipoprotein particles (sHDLP). All concentrations are expressed in µM. Ketone body concentration was defined as the sum of BHB, AcAc, and acetone.

The MMX biomarker contains contributions from valine, leucine, isoleucine, and citrate concentrations. The sHDLP (HDL particle size < 9 nm) and GlycA, an inflammation marker corresponding to glycan residues of acute-phase glycoproteins, contribute to the IVX biomarker. MVX is a biomarker that combines MMX and IVX. The IVX, MMX, and MVX were computed using algorithms developed by Otvos et al. [12].

IVX, MMX, and MVX were computed using the following formulas:

IVX=9−0.0027 GlycA−0.46079 sHDLP+0.0006325 GlycA×sHDLP

$${IVX}_{min}=2.0⟹score=1$$

$${IVX}_{max}=8.3⟹score=100$$

$$MMX=\left(0.75097\left[4-0.02234\;Leu+{0.0000528\;Leu}^{2}\right]\right)+\;\left(0.55737\left[7-0.02895\;Val+0.0000608\;{Val}^{2}\right]\right)+\left(0.00867\;Ile\right)+\;\left(0.65649[1+0.0025\;Cit+0.0000167\;{Cit}^{2}]\right)$$

$${MMX}_{min}=1.281⟹score=1$$

$${MMX}_{max}=2.0⟹score=100$$

MVX=2.72923 IVX+11.96062 lnMMX−1.12749 IVX×lnMMX

$${MVX}_{min}=20.3⟹score=1$$

$${MVX}_{max}=28.0⟹score=100$$

In the formulas, Leu, Val, Ile, and Cit refer to leucine, valine, isoleucine, and citrate levels. The subscripts min and max refer to the minimum and maximum values of the corresponding index.

### 2.3. Lipids and Apolipoproteins

Total cholesterol (TC), high-density lipoprotein cholesterol (HDL-C), and triglycerides (TG) measurements were obtained from a standard, non-fasting lipid profile, and the apolipoproteins (Apo) Apo-AI, Apo-AII, ApoB, and ApoE were obtained using apolipoprotein profiling. Our previously published [13] methods for lipid and apolipoprotein analyses were used. Analysts were blinded to the clinical status of samples.

TC, HDL-C, and triglycerides (TG) were measured with diagnostic reagent kits (Sekisui Diagnostics, Lexington, MA, USA) on an ABX Pentra 400 (Horiba Instruments, Irvine, CA, USA) automated chemistry analyzer. Apolipoproteins (Apo) Apo-AI, Apo-AII, ApoB, and ApoE were measured with immunoturbidometric diagnostic kits (Kamiya Biomedical, Thousand Oaks, CA, USA). Low-density lipoprotein cholesterol (LDL-C) was computed from TC, TG, and HDL-C using the Friedewald equation [14].

### 2.4. Lipid Peroxidation Products and Antioxidant Defense Enzyme Activity Measurements

Lipid peroxidation products and a panel of antioxidant defense enzyme activities were measured in a subset of 96 participants, consisting of 24 HC-24, 52 RRMS, and 20 PMS.

**Lipid Peroxidation Products:** We measured the major hydroxy (HO) and hydroperoxy (Hp) lipid peroxidation products of octadecadienoic (ODE, 18:2 or linoleic acid), octadecatrienoic (OTE, 18:3 or linolenic acid), and eicosatetraenoic (ETE, 20:4 or arachidonic acid) fatty acids species. The lipid peroxidation products quantitated include 9S-hydroperoxy-10Z,12E octadecadienoic acid (9-HpODE), 13-HpODE, 9-HODE, 13-HODE, 13-HOTE, 12-HpETE, 12-HEPE, 12-HETE, and 5-HETE.

The LC-MS sample preparation and assay are described in the Supplementary File.

**Antioxidant Defense Enzyme Activities:** The activities of antioxidant enzymes superoxide dismutase (SOD), glutathione peroxidase (GPX), glutathione reductase (GSHR), glutathione-S-transferase (GST), and paraoxonase 1 (PON1) were measured as previously described [15] and are detailed in the Supplementary File.

### 2.5. Data Analysis

The R statistical computing program was utilized for all statistical analyses; results were visualized using the *ggplot2* package in R (4.1.2).

Ketone bodies, lipid peroxidation products, and ambulation time were logarithm (base 10) transformed for regression analysis. EDSS quartiles were created using the *ntile* function: Quartile 1: 0 to EDSS < 2; Quartile 2: 2 ≤ EDSS ≤ 3; Quartile 3: 3 < EDSS ≤ 5.5; Quartile 4: EDSS > 5.5.

For Table 1, the differences in the frequencies of females and males and disease-modifying therapies (DMT) for the HC-RR-PMS status variables were assessed with the $\chi^{2}$ test. Age and BMI differences were evaluated with a one-way ANOVA. The EDSS differences between PMS and RR-MS groups were evaluated using the Mann-Whitney U test. The disease duration and age differences between PMS and RR-MS groups were evaluated with the independent samples *t*-test.

**Figure 2:**
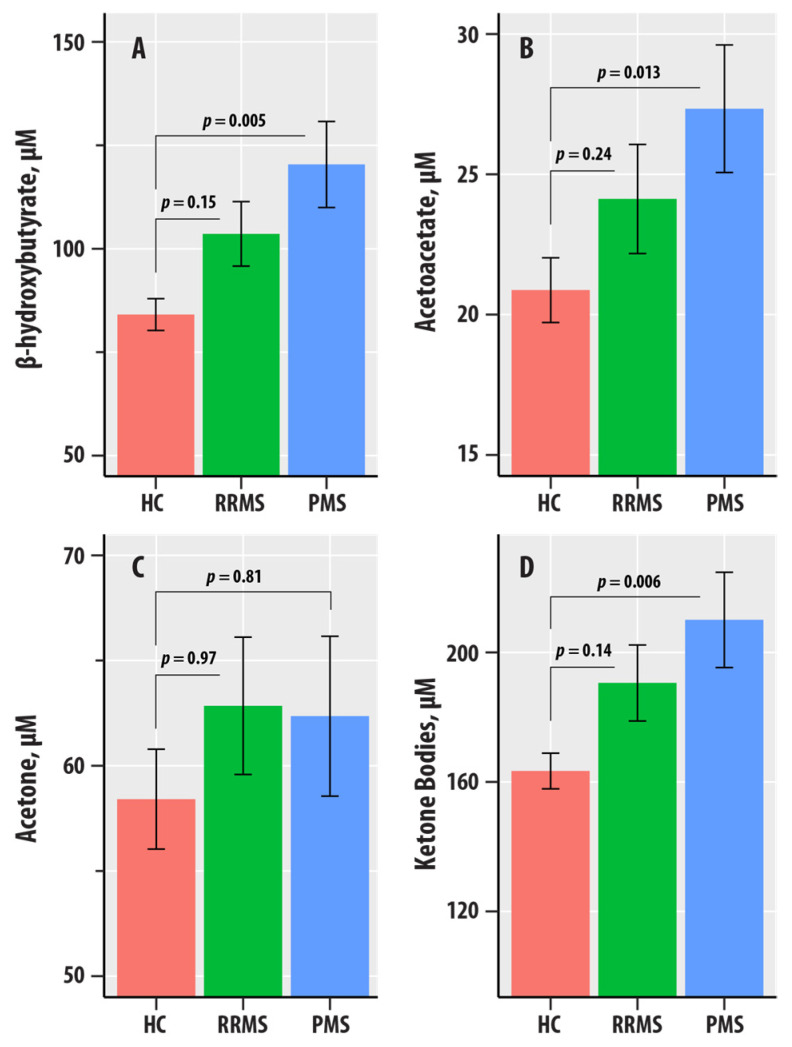
(**A**–**D**) are bar graphs summarizing results from nuclear magnetic resonance spectroscopic profiling in healthy controls (HC, salmon bars), relapsing-remitting multiple sclerosis (RRMS, green bars), and progressive MS (PMS, blue bars). The levels of β-hydroxybutyrate (**A**), acetoacetate (AcAc, (**B**)), acetone (**C**), and total ketone bodies (**D**) are in µM. The bars represent mean values, and the error bars are standard errors. The *p*-values corresponding to the differences in slopes of the RR-MS and PMS groups relative to the HC group reference were obtained from the regression summary.

In the linear regression analyses for Figure 2, the logarithm-transformed values of the individual ketone body biomarkers (BHB, AcAc, acetone, or ketone bodies) were dependent variables; age, sex, BMI, and HC-RR-PMS status were included as predictors.

Inflammatory vulnerability index (IVX), metabolic-malnutrition index (MMX), and metabolic vulnerability index (MVX) were individually analyzed as dependent variables in linear regression analyses for Table 2. Lipids and apolipoproteins, alanine, valine, leucine, isoleucine, citrate, small HDL particles (sHDLP), and GlycA were each assessed as dependent variables in linear regression analyses for Table 3. EDSS, EDSS quartiles, MSSS, and logarithm (base 10) of ambulation time were each assessed as dependent variables in linear regression analyses for Table 4. In all these regression analyses, the individual ketone body biomarkers (the logarithm-transformed values of BHB, AcAc, acetone, or ketone bodies), age, sex, BMI, and HC-RR-PMS status were included as predictors.

The regression results were summarized as the regression coefficient for the ketone body biomarker β, generalized eta-squared effect size measure $\eta^{2}$, and the predictor *p*-value.

## 3. Results

### 3.1. Clinical and Demographic Characteristics

The clinical and demographic information for the HC, RRMS, and PMS groups are shown in Table 1. Patients with PMS had a greater EDSS (6.0 vs. 2.5, *p* < 0.001, Mann-Whitney U test), a longer disease duration (21.5 vs. 12.0 years, *p* < 0.001, independent samples *t*-test) and were overall older (54.1 vs. 44.2 years, *p* < 0.001, independent samples *t*-test) when compared to the RRMS group. These differences are representative of the underlying MS disease course.

### 3.2. Associations of Ketone Bodies with MS Disease Course

Figure 2 summarizes the levels of BHB, AcAc, acetone, and ketone bodies in the HC, RR, and PMS groups. In regression adjusted for age, sex, BMI, and HC-RR-PMS status, BHB ($\eta^{2}$ = 0.020, p = 0.018), AcAc ($\eta^{2}$ = 0.015, p = 0.046), and ketone bodies ($\eta^{2}$ = 0.019, p = 0.021) were associated with the HC-RR-PMS status variable. The *p*-values corresponding to the differences in slopes of the RR-MS and PMS groups relative to the HC group reference were obtained from the regression summary. The levels of BHB (*p* = 0.005), AcAc (*p* = 0.013), and ketone bodies (*p* = 0.006) were higher in PMS compared to HC. We did not obtain evidence for differences in the RR-MS vs. HC comparisons for BHB, AcAc, acetone, and ketone bodies.

### 3.3. Associations of Ketone Bodies with Inflammatory and Metabolic Vulnerability Indices

Table 2 summarizes the association results for IVX, MMX, and MVX with the ketone bodies biomarkers. The regression analyses were adjusted for age, sex, BMI, and HC-RR-PMS status.

**Figure 3:**
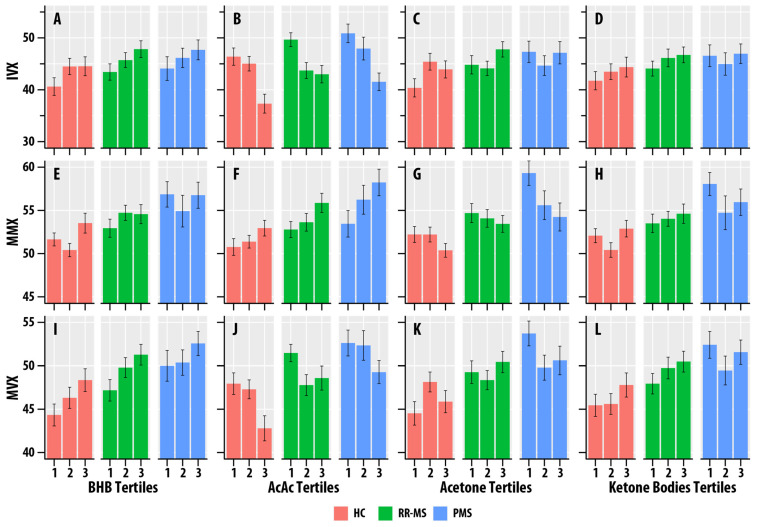
(**A**–**L**). Faceted tertile bar graphs summarizing results for inflammatory vulnerability index (IVX, (**A**–**D**)), metabolic-malnutrition index (MMX, (**E**–**H**)), and metabolic vulnerability index (MMX, (**I**–**L**)) in healthy controls (HC, salmon bars), relapsing-remitting multiple sclerosis (RR, green bars), and progressive MS (PMS, blue bars). The *x*-axis corresponds to the tertiles of β-hydroxybutyrate (BHB, (**A**,**E**,**I**)), acetoacetate (AcAc, (**B**,**F**,**J**)), acetone (**C**,**G**,**K**), and total ketone bodies (KB, (**D**,**H**,**L**)). The bars represent mean values, and the error bars are standard errors.

BHB was positively associated with IVX ($\eta^{2}$ = 0.012, p = 0.029), MMX ($\eta^{2}$ = 0.023, p = 0.002), and MVX ($\eta^{2}$ = 0.034, p < 0.001). AcAc was positively associated with MMX ($\eta^{2}$ = 0.035, p < 0.001), but negatively associated with IVX ($\eta^{2}$ = 0.073, p < 0.001) and MVX ($\eta^{2}$ = 0.025, p = 0.002). Acetone was positively associated with IVX ($\eta^{2}$ = 0.015, p = 0.014). Total ketone body concentration was positively associated with MMX ($\eta^{2}$ = 0.014, p = 0.019) and MVX ($\eta^{2}$ = 0.013, p = 0.023). Figure 3 summarizes the associations of MMX, IVX, and MVX for the tertiles of BHB, AcAc, acetone, and ketone bodies. The higher tertiles of BHB and ketone bodies correlated with greater MMX, IVX, and MVX values. The higher tertiles of AcAc were associated with lower MVX and IVX values and higher MMX values.

### 3.4. Lipid and Amino Acid Metabolic Pathways and Ketone Bodies

We examined ketone bodies’ associations with lipid and amino acid metabolism biomarkers and glycation burden (GlycA).

We did not obtain evidence for associations of BHB, AcAc, acetone, and ketone bodies with TG, TC, LDL-C, ApoB, or ApoE (Table 3). HDL-C was positively associated with ketone bodies ($\eta^{2}$ = 0.013, p = 0.020) and exhibited association trends with BHB (p = 0.063) and AcAc (p = 0.077). Apo-AI, the characteristic apolipoprotein of HDL, was positively associated with BHB ($\eta^{2}$ = 0.014, p = 0.019) and ketone bodies ($\eta^{2}$ = 0.015, p = 0.014). Apo-AII was associated with BHB ($\eta^{2}$ = 0.012, p = 0.027) and AcAc ($\eta^{2}$ = 0.011, p = 0.033).

**Figure 4:**
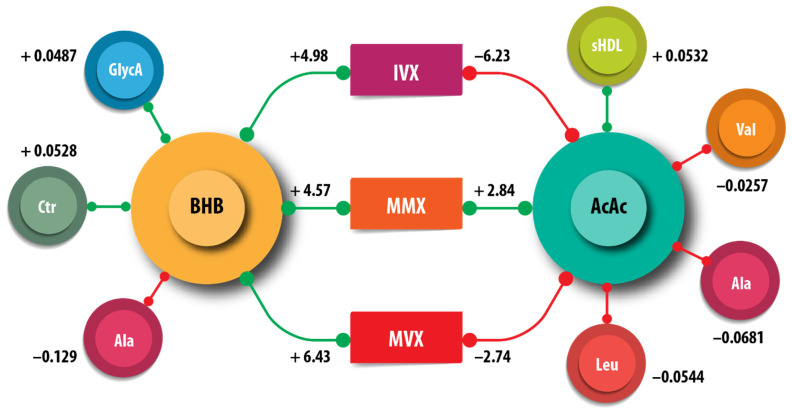
Schematic summary of the associations of β-hydroxybutyrate (BHB) and acetoacetate (AcAc), with the metabolites citrate (Cit), alanine (Ala), leucine (Leu), small high-density lipoprotein particle (sHDLP), and GlycA. The inflammatory vulnerability index (IVX), metabolic malnutrition index (MMX), and metabolic vulnerability index (MVX) are also shown. The numbers indicate the regression coefficients from Table 3. The green lines correspond to positive coefficients, whereas the red lines indicate negative coefficients.

We examined the associations of the amino acids, citrate, small HDL particles (sHDLP), and GlycA measured by NMR with ketone bodies. Acetoacetate, but not BHB, was positively associated with sHDLP (AcAc $\eta^{2}$ = 0.081, p = <0.001) (Table 3). The amino acids alanine, valine, and leucine were negatively associated with AcAc; Alanine was additionally negatively associated with BHB. Citrate and GlycA were positively associated with BHB. Leucine was positively associated with acetone. We did not obtain evidence for associations of the ketone bodies with isoleucine. These associations are summarized in Figure 4.

### 3.5. Lipid Peroxidation Products and Antioxidant Defense Enzyme Activities

Lipid peroxidation products and the antioxidant defense enzymes SOD, GPX, GSHR, GST, and PON1 were measured in a subset of *n* = 96 participants with *n* = 24 HC, *n* = 52 RRMS, and *n* = 20 PMS subjects. The mean age (46.1 years for HC, 44.3 years for RR, and 55.4 years for PMS) and gender distribution (62.5%, 73%, and 80% female for HC, RR, and PMS groups, respectively) were similar to the larger set with ketone bodies in Table 1.

We did not obtain evidence of associations between any of the lipid peroxidation products and BHB, AcAc, acetone, or ketone bodies (Supplementary Table S1).

BHB was positively associated with GPX ($\eta^{2}$ = 0.051, *p* = 0.038, Supplementary Table S1). We did not obtain evidence for any other associations of BHB, AcAc, acetone, and ketone bodies with any of the antioxidant defense enzyme activities measured (Supplementary Table S1). The underlying mechanisms for the isolated association of BHB with GPX are unknown.

### 3.6. MS Disability and Ketone Bodies

Table 4 displays EDSS, MSSS, and ambulation time regression results with ketone bodies adjusted for age, sex, and BMI.

**Figure 5:**
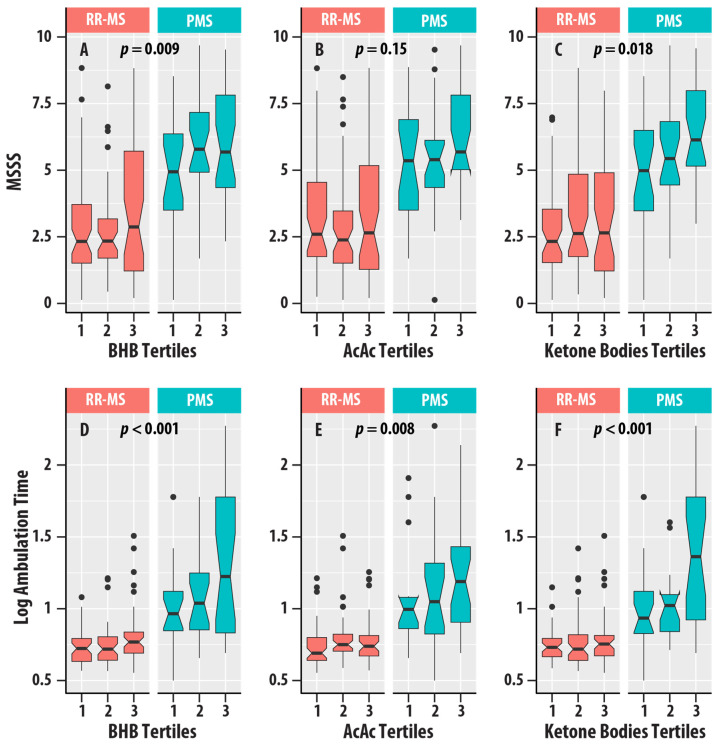
(**A**–**C**) are box plots of MS Severity Score (MSSS) with tertiles of β-hydroxybutyrate (BHB, (**A**)), acetoacetate (AcAc, (**B**)), and total ketone bodies (**C**). (**D**–**F**) are box plots of logarithm (base 10) ambulation time with tertiles of β-hydroxybutyrate (BHB, (**D**)), acetoacetate (AcAc, (**E**)), and total ketone bodies (**F**). Relapsing-remitting MS (RR-MS) is shown in salmon boxes, and progressive MS (PMS) is shown in teal boxes. The horizontal lines are medians, the upper and lower edges of the box are the 25th and 75th percentiles, the error bars are the 5th and 95th percentiles, and the circles are outliers. The *p*-values for the ketone bodies variable from regression analyses (Table 4) are shown.

Higher EDSS scores (EDSS > 5.5) were associated with higher concentrations of BHB ($\eta^{2}$ = 0.021, p = 0.022), AcAc ($\eta^{2}$ = 0.016, p = 0.048), and ketone bodies ($\eta^{2}$ = 0.017, p = 0.041). MSSS was positively associated with BHB ($\eta^{2}$ = 0.031, p = 0.009) and ketone bodies ($\eta^{2}$ = 0.026, p = 0.018). Ambulation time was positively associated with BHB ($\eta^{2}$ = 0.065, p < 0.001), AcAc ($\eta^{2}$ = 0.036, p = 0.008), and ketone bodies ($\eta^{2}$ = 0.058, p < 0.001). Figure 5 summarizes the distributions of EDSS, MSSS, and ambulation time for the different tertiles of ketone bodies in the RR-MS and PMS groups.

## 4. Discussion

We found increases in acetoacetate and BHB levels in MS vs. HC and associations with greater MSSS and ambulation time. Increases in acetoacetate and BHB in MS were also reported in an NMR-based metabolomic study by Cocco et al. [11]. AcAc was positively associated with IVX and MVX and negatively associated with MMX, and BHB was negatively associated with IVX, MMX, and MVX. Alanine and valine were associated with reduced levels of AcAc.

Ketogenesis is a process that occurs primarily in the liver and produces the ketone bodies (acetoacetate, BHB, and acetone) from excess acetyl-CoA under conditions of low carbohydrate and high fatty acid levels. Increases in ketone bodies occur when fatty acid oxidation is active, e.g., diabetes and starvation, and can fuel the energy needs of the brain and heart when glucose is scarce. Fatty acid oxidation and mitochondrial metabolism are essential for M2 macrophages and memory T and B cells [16].

We analyzed ketone bodies in serum samples collected under non-fasting conditions because fasting can enhance ketone body production. Therefore, our ketone body measurements likely reflect typical concentrations present in MS patients, and the increases we found in the MS group could be mediated by metabolic stress. The associations of ketone body concentrations with MMX and MVX are consistent with this mechanism. Additionally, we found decreases in leucine, a ketogenic amino acid important in metabolic regulation, that mirrored the decreases in AcAc.

A strength of our study is the availability of ambulation and disability data, including the EDSS, MSSS, and timed ambulation. Higher concentrations of BHB, AcAc, and total ketone bodies were associated with worsened ambulation time, which evaluates leg function and mobility in pwMS. Timed ambulation can detect subtle differences in disability, even in patients who are minimally affected. MSSS, which was positively correlated with BHB and total ketone bodies, adjusts the EDSS for disease duration and has been shown to predict disability accrual over time. The impact of endogenous ketone bodies on physical disability in MS has not previously been characterized and could be a useful framework for understanding the interactions between metabolism and MS disability.

Our findings underscore the connection between ketone bodies and lipid and amino acid metabolism. The availability of validated metrics such as IVX, MMX, and MVX allowed us to assess interactions among various macronutrient metabolic pathways. IVX measures chronic inflammatory processes and includes contributions from sHDLP and GlycA, an NMR signature produced by glycated acute phase proteins. MMX includes a linear term for isoleucine and non-linear contributions from leucine, valine, and citrate. MVX incorporates linear terms from IVX and MMX, and an interaction term that includes both IVX and MMX. We found that ketone body levels were associated with IVX, MMX, and MVX. However, BHB and AcAc exhibited opposing effects on IVX and MVX, showing positive associations with BHB and negative associations with AcAc. To our knowledge, the interdependencies of ketone bodies with inflammatory processes and metabolic vulnerability in MS remain relatively unexplored.

A limitation of our study is that we did not have follow-up data on ketone bodies, which would have provided additional information on the trajectory of ketone bodies during MS progression. While we had data on lipids, we did not have fasting glucose and insulin levels, which could assess if the increases in ketone bodies are more strongly or weakly dependent on low glucose levels in MS than controls. Fasting glucose and insulin levels can also be used to obtain homeostatic model assessment of insulin resistance (HOMA-IR) and homeostatic model assessment of beta-cell function (HOMA-BETA), which are indices for evaluating the inter-dependence of glucose homeostasis with ketone bodies. High HOMA-IR indicates insulin resistance. A low HOMA-BETA value indicates reduced beta cell function and poor glucose tolerance and predicts type II diabetes. Additionally, we did not consider lifestyle factors such as exercise and dietary interventions. Endurance exercises can increase ketone body production. While exercise is beneficial in managing and improving MS symptomology, most patients will reduce their physical activity due to disability, lack of stability and muscle strength, and loss of fitness.

Glial cells have some capacity for ketogenesis and can fuel neurons [5,17]. Interestingly, we found that alanine, valine, and leucine were negatively associated with ketone bodies but did not obtain evidence for associations with isoleucine. Leucine is a ketogenic amino acid that is catabolized into acetyl-CoA and acetoacetate to enter the citric acid cycle. Alanine and valine are glucogenic amino acids and are catabolized to glucose, and isoleucine is amphibolic. The leucine sensor SAR1B enables leucine levels to potently activate the mammalian target of rapamycin complex-1 (mTORC1) [18]. mTORC1 is a master regulator of nutrient sensing that can stimulate protein and cholesterol synthesis and inhibit ketogenesis [19,20]. Appropriate mTORC1-dependent signaling is essential for the synthesis of myelin lipids and proteins in oligodendrocytes [21]. mTORC1 signaling has been implicated in MS pathology via its effects on microglia, astrocytes, and oligodendrocytes and mechanisms including cell cycle control, protein synthesis, cellular senescence, and other biological processes [22,23].

In recent years, dietary interventions have been investigated to ameliorate MS disability and neurodegeneration [24,25]. AcAc, BHB, and acetone are increased in pwMS compared to healthy controls, contributing to an altered energy metabolism [11]. Ketogenic diet studies in MS patients have reported improvements in physical, mental, and neurological health in those with MS over 6 months [24,25]. However, long-term ketogenic dieting has been linked to higher concentrations of LDL cholesterol and a 2-fold higher risk of adverse cardiac events [26]. Healthy rodents on a ketogenic diet chow showed induced senescence, metabolic acidosis, and increased oxidative stress in normal tissues [27,28]. Additionally, MRI spectroscopic markers collected on a cohort of rats on long-term ketogenic diets showed structural and functional changes [29]. However, MRI research in humans is lacking.

## 5. Conclusions

In conclusion, our results demonstrate alterations of ketone bodies orchestrated with associations with inflammatory and metabolic vulnerability in MS patients. Our findings suggest that amino acids such as leucine and HDL particles may be involved in modulating ketone bodies in MS.

## Figures and Tables

**Table 1:** Demographic and clinical characteristics at baseline and follow-up.

	HC	RR-MS	P-MS	*p*-Value
**Sample size *n***	152	184	91	
**Gender, Female (%)**	85 (55.9)	139 (75.5)	66 (72.5)	<0.001
**Age, years**	45.7 (13.9)	44.2 (9.70)	54.1 (8.82)	<0.001
**Body mass index, kg/m^2^**	28.1 (5.97)	27.0 (6.09)	25.6 (3.30)	0.009
**Race:**				0.48
Caucasian	131 (87.9%)	168 (92.3%)	86 (94.5%)	
African American	13 (8.7%)	9 (4.95%)	4 (4.4%)	
Hispanic/Latino	1 (0.67%)	3 (1.65%)	1 (1.1%)	
Asian	3 (2.0%)	1 (0.55%)	0	
Other	1 (0.67%)	0	0	
Missing	3 (2.0%)	3 (1.65%)	0	
**Disease duration, years**		12.0 (8.49)	21.5 (11.4)	<0.001
**EDSS**		2.5 (1.5–3.5)	6.0 (5.0–6.5)	<0.001
**Disease-modifying treatments:**				0.17
No treatment	-	20 (10.9%)	16 (17.6%)	
Interferon	-	66 (35.7%)	31 (34.1%)	
Glatiramer acetate	-	34 (18.5%)	25 (27.5)	
Other	-	43 (23.4%)	16 (17.6%)	
Missing	-	21 (11.4%)	3 (3.3%)	
**Ketone bodies**				
Ketone bodies, µM	163 (67.8)	191 (159)	210 (140)	0.021
β-hydroxybutyrate, µM	84.1 (47.3)	104 (106)	120 (99)	0.018
Acetoacetate, µM	20.9 (14.2)	24.1 (26.4)	27.3 (21.7)	0.046
Acetone, µM	58.4 (29.3)	62.8 (44.3)	62.4 (36.2)	0.96

HC: Healthy controls; RRMS: Relapsing-remitting multiple sclerosis; PMS: Progressive multiple sclerosis; BMI: Body mass index; EDSS: Expanded disability status scale.

**Table 2:** Regression results for the associations of ketone bodies with inflammatory vulnerability index (IVX), metabolic-malnutrition index (MMX), and metabolic vulnerability index (MVX). The ketone bodies (in µM) were logarithm (base 10) transformed and the regression was adjusted for age, sex, body mass index, and HC-RR-PMS status. The regression coefficient for the ketone body biomarker β, generalized eta-squared effect size measure $\eta^{2}$, and the predictor p-value are shown.

Biomarker	IVX	MMX	MVX
BHB	4.98 0.012 (0.029)	4.57 0.023 (0.002)	6.43 0.034 (< 0.001)
AcAc	−6.23 0.073 (<0.001)	2.84 0.035 (<0.001)	−2.74 0.025 (0.002)
Acetone	5.41 0.015 (0.014)	−2.15 0.005 (0.14)	2.51 0.006 (0.13)
Ketone bodies	3.01 0.003 (0.30)	4.54 0.014 (0.019)	5.03 0.013 (0.023)

BHB: β-hydroxybutyrate; AcAc: Acetoacetate; IVX: Inflammatory vulnerability index; MMX: Metabolic-malnutrition index; MVX: Metabolic vulnerability index.

**Table 3:** Regression analyses of ketone bodies vs. lipids and apolipoproteins, alanine, valine, leucine, isoleucine, citrate, small HDL particles, and GlycA adjusted for age, sex, body mass index, and HC-RR-PMS status. The regression coefficient for the ketone body biomarker β, generalized eta-squared effect size measure $\eta^{2}$, and the predictor *p*-value are shown.

Lipid Biomarker	BHB	Acetoacetate	Acetone	Ketone Bodies
Triglycerides	−4.77 <0.001 (0.74)	0.489 <0.001 (0.94)	−6.45 <0.001 (0.64)	−17.4 0.002 (0.34)
Total cholesterol	12.7 0.006 (0.11)	4.67 0.003 (0.24)	9.66 0.004 (0.20)	16.3 0.007 (0.11)
HDL-C	5.31 0.009 (0.063)	2.53 0.008 (0.077)	2.70 0.002 (0.32)	8.48 0.013 (0.020)
LDL-C	1.55 <0.001 (0.78)	−3.13 0.003 (0.26)	8.1 0.006 (0.12)	3.3 <0.001 (0.64)
Apo-AI	10.5 0.014 (0.019)	−0.637 <0.001 (0.78)	8.18 0.009 (0.058)	14.1 0.015 (0.014)
Apo-AII	2.78 0.012 (0.027)	−1.35 0.011 (0.033)	1.50 0.004 (0.22)	2.76 0.007 (0.086)
ApoB	0.985 <0.001 (0.83)	-2.00 0.002 (0.38)	2.90 0.001 (0.5)	0.476 <0.001 (0.93)
ApoE	−227 0.007 (0.086)	16.6 <0.001 (0.8)	26.2 <0.001 (0.84)	−177 0.003 (0.30)
**NMR Biomarker**	**BHB**	**Acetoacetate**	**Acetone**	**Ketone Bodies**
Alanine	−0.129 0.105 (<0.001)	−0.0681 0.116 (<0.001)	−0.0296 0.006 (0.12)	−0.172 0.115 (<0.001)
Valine	−0.0141 0.001 (0.49)	−0.0257 0.015 (0.013)	0.0326 0.007 (0.099)	−0.00641 <0.001 (0.81)
Leucine	−0.00259 <0.001 (0.93)	−0.0544 0.032 (<0.001)	0.105 0.033 (<0.001)	0.0240 <0.001 (0.54)
Isoleucine	0.0191 0.001 (0.48)	−0.0227 0.007 (0.099)	0.0271 0.003 (0.30)	0.0177 <0.001 (0.61)
Citrate	0.0528 0.01 (0.049)	0.0196 0.005 (0.15)	0.0383 0.005 (0.14)	0.0811 0.014 (0.018)
sHDLP	−0.000333 <0.001 (0.99)	0.0532 0.081 (<0.001)	−0.0471 0.017 (0.008)	0.00907 <0.001 (0.70)
GlycA	0.0487 0.03 (<0.001)	−0.00788 0.003 (0.26)	−0.00249 <0.001 (0.85)	0.0347 0.009 (0.053)

BHB: Beta-hydroxybutyrate; HC-RR-PMS: HC: Healthy controls; RR: relapsing-remitting; PMS: progressive MS; HDL-C: High-density lipoprotein cholesterol, LDL-C: Low-density lipoprotein cholesterol; Apo: Apolipoprotein; NMR: Nuclear magnetic resonance; sHDLP: Small high-density lipoprotein particles.

**Table 4:** Regression results for the associations of EDSS, EDSS quartiles, MSSS, and logarithm (base 10) of ambulation time. The ketone bodies (in µM) were logarithm (base 10)-transformed. The regression analyses were adjusted for age, sex, and body mass index. The regression coefficient for the ketone body biomarker β, generalized eta-squared effect size measure $\eta^{2}$, and the predictor *p*-value are shown.

Biomarker	EDSS	EDSS Quartiles	MSSS	Ambulation Time
BHB	0.915 0.015 (0.051)	0.566 0.021 (0.022)	1.54 0.031 (0.009)	0.283 0.065 (<0.001)
AcAc	0.469 0.01 (0.12)	0.315 0.016 (0.048)	0.549 0.01 (0.15)	0.135 0.036 (0.008)
Acetone	0.24 0.001 (0.59)	0.0736 0.00041 (0.75)	0.331 0.002 (0.55)	0.0822 0.006 (0.29)
Ketone bodies	1.06 0.013 (0.071)	0.634 0.017 (0.041)	1.74 0.026 (0.018)	0.335 0.058 (<0.001)

BHB: β-hydroxybutyrate; AcAc: Acetoacetate; EDSS Quartile 1: 0 to EDSS < 2; EDSS Quartile 2: 2 ≤ EDSS ≤ 3; EDSS Quartile 3: 3 < EDSS ≤ 5.5; EDSS Quartile 4: EDSS > 5.5.

## Data Availability

The data that support the findings of this study are available upon reasonable request from the principal investigator of the clinical study (Robert Zivadinov). The data is not publicly available because it contains information that could compromise the privacy of research participants.

## Supplementary Materials

The following supporting information can be downloaded at: https://www.mdpi.com/article/10.3390/nu17040640/s1, Table S1. Regression analyses of ketone bodies vs. lipid peroxidation markers, lipids and apolipoproteins, and antioxidant defense enzymes adjusted for Age, Sex, BMI, and HC_RR_PMS status. Results are summarized as eta squared ($\eta^{2}$), and *p*-value. References [30,31,32,33] are cited in the Supplementary Materials.
