# Supplementary material for: Endogenous Ketone Bodies Are Associated with Metabolic Vulnerability and Disability in Multiple Sclerosis
Source: Nutrients. 2025 Feb 11;17(4):640. doi: 10.3390/nu17040640 (PMC11858685; doi:10.3390/nu17040640)
Supplement: Supplementary file 1 [file nutrients-17-00640-s001.zip › nutrients-3450981-supplementary.pdf]

SUPPLEMENTARY FILE

Ketone Bodies in Multiple Sclerosis

Taylor R. Wicks [1], Anna Wolska [2], Irina Shalaurova [3], Richard W. Browne [4], Bianca Weinstock-Guttman [5], Robert Zivadinov [6, 7], Alan T. Remaley [2], James Otvos [2, 4], Murali Ramanathan [1, 5]

Departments of Pharmaceutical Sciences [1], Biotechnical and Clinical Laboratory Sciences [4], Neurology [5], Buffalo Neuroimaging Analysis Center [6], Center for Biomedical Imaging at Clinical Translational Science Institute [7], University at Buffalo, The State University of New York, Buffalo, NY, USA.

[2] Lipoprotein Metabolism Laboratory, National Heart, Lung and Blood Institute, National Institutes of Health, Bethesda, MD, USA.

[3] LabCorp Diagnostics, Morrisville, NC, USA.

CORRESPONDING AUTHOR: Murali Ramanathan

355 Pharmacy, Department of Pharmaceutical Sciences

University at Buffalo, The State University of New York

Buffalo, NY 14214-8033.

Phone: (716)-645-4846. E-mail Murali@Buffalo.Edu

Running Head: Ketone Bodies in MS

Keywords: Ketone Bodies, beta-hydroxybutyrate, acetoacetate, lipid peroxidation, antioxidant defense

SUPPLEMENTARY METHODS

Lipid Peroxidation Products and Antioxidant Defense Enzyme Activity Measurements

Lipid peroxidation products and a panel of antioxidant defense enzyme activities were measured in a subset of n = 96 participants consisting of 24 HC-24, 52 RRMS, and 20 PMS.

Lipid Peroxidation Products: We measured the major hydroxy (H) and hydroperoxy (Hp) lipid peroxidation products of octadecadienoic (ODE, 18:2 or linoleic acid), octadecatrienoic (OTE, 18:3 or linolenic acid) and eicosatetraenoic (ETE, 20:4 or arachidonic acid) fatty acids species as previously described (30).

Hydroxy and hydroperoxy fatty acid standards ((±)-9-hydroxy-10E,12Z-octadecadienoic acid (9-HODE), (±)-13-hydroxy-9Z,11E-octadecadienoic acid (13-HODE), 9-hydroperoxy-10E,12Z-octadecadienoic acid (9-HpODE), (±)13-hydroperoxy-9Z,11E-octadecadienoic acid (13-HpODE), 13S-hydroxy-9Z,11E,15Z-octadecatrienoic acid (13(s)-HOTrE), (±)5-hydroxy-6E,8Z,11Z,14Z-eicosatetraenoic acid (5-HETE), (±)12-hydroxy-5Z,8Z,10E,14Z-eicosatetraenoic acid (12-HETE)), and deuterated internal standards (9S-hydroxy-10E,12Z-octadecadienoic-9,10,12,13-d4 acid (9(s)-HODE-d4) and 12S-hydroxy-5Z,8Z,10E,14Z-eicosatetraenoic-5,6,8,9,11,12,14,15-d8 acid (12(s)-HETE-d8)) were purchased from Cayman Chemical (Ann Arbor, MI). Cholesterol esterase (CE) was obtained from Millipore-Sigma from Pseudomonas fluorescence. The CE enzyme was prepared at a concentration of 20 U/mL in cholic acid and Triton buffer (CaT buffer) containing 0.1M potassium phosphate, 0.05 M sodium chloride, 5 mM cholic acid, and 0.1% Titon X-100 at pH 7.4. Aliquots were frozen at -80°C (31).

Total fatty acids were hydrolyzed from lipid esters using cholesterol esterase (CE) (31). Plasma samples (200 μL), 10 μl of 0.1 g/L of ethanolic butylated hydroxytoluene (0.005%), 100 μL CE in CaT buffer (total of 2 units CE per sample), and 100 μL additional CaT buffer were incubated at 30°C for 20 minutes. Following incubation, 20 μL Internal Standard Reagent (9-HODE-d4 and 12-HETE-d8 each at 20 μg/mL in isopropyl alcohol) and 1.0 mL 10%v/v acetic acid in water/2-propanol/hexane (2/20/30, v/v/v) were added and the sample was vortexed. Analytes were extracted with 2.0 mL of hexane and evaporated under nitrogen at 37°C. The dry extract was dissolved into 200 μl of 85% v/v methanol in water, filtered through a SPIN-X 0.22 μm microcentrifuge filter, and transferred to an HPLC autosampler vial with a limited volume conical vial insert.

LC-MS analysis was conducted on a Shimadzu Scientific (Columbia, MD) LCMS-2010A mass spectrometry system with electrospray ionization in negative ion mode. Mobile phase A composition was 100% water with 10 mM ammonium acetate and 0.1% formic acid, and mobile phase B was 70:30 Acetonitrile:2-propanol (v/v) with 10 mM ammonium acetate and 0.1% formic acid. The mobile phase gradient was 40% B for 10 min, linear increase to 55% A for 15 min, wash at 100% B for 5 min, followed by re-equilibration at 40% B for 5 min; total run time was 35 min. The column was a Waters Acquity BEH-C18 2.1 × 50 mm, 1.7 μm UPLC column at 60 °C, and a flow rate of 0.25 mL/min. Data were acquired in selected ion monitoring (SIM) mode for maximum sensitivity. Each analyte was detected as its [M-H]- ion. Internal standard 12-HETE-d8 was used for the quantification of all eicosanoid analytes. Internal standard 9-HODE-d4 was used for the quantitation of all other analytes. For the ODE and OTE-derived LPO products, the limit of detection (LOD) is 5 pmol, and the LOD for the

ETE-derived LPO products is 15.3 pmol. Assay coefficients of variation (CV) are 4.7–12%, and recoveries are 87–103% (30-33).

Antioxidant Defense Enzyme Activity: The antioxidant enzymes superoxide dismutase (SOD), glutathione peroxidase (GPX), glutathione reductase (GSHR), and glutathione-S-transferase (GST) were measured utilizing kinetic enzyme assay on a Cobas MIRA automated chemistry analyzer (32) as previously described (15). Overall, SOD activity was defined as the inhibition of oxidation of cytochrome C by xanthine/xanthine oxidase under normal conditions, while one unit of SOD activity was an induction of 50% inhibition within the reaction. GPX and GSHR activity was validated using single lot concentrations of OxiTek reagent kits. GST activity was determined by the reaction rate between 1-chloro-2,4-dinitrobenzene and reduced glutathione.

Paraoxonase-1 (PON1) activities were determined using phenyl acetate as a substrate for the arylesterase (ARYL) and paraoxon (diethyl 4-nitrophenyl phosphate) for paraoxonase (PXON) activities of PON1 (33). The assay CVs are 0.6 to 1.4% on the Cobas MIRA automated chemistry analyzer (33).

SUPPLEMENTARY TABLES

Table S1. Regression analyses of ketone bodies vs. lipid peroxidation markers, lipids and apolipoproteins, and antioxidant defense enzymes adjusted for Age, Sex, BMI, and HC_RR_PMS status. Results are summarized as eta squared (η^2), and *p*-value.

	BHB	Acetoacetate	Acetone	Ketone Bodies
**Lipid peroxides**				
9-HODE	0.001 (0.74)	0.026 (0.15)	0.002 (0.68)	< 0.001 (0.86)
13-HODE	0.001 (0.76)	0.01 (0.37)	< 0.001 (0.94)	< 0.001 (0.94)
9-HpODE	0.003 (0.73)	0.009 (0.40)	0.002 (0.69)	< 0.001 (0.89)
13-HpODE	< 0.001 (0.86)	0.008 (0.42)	< 0.001 (1.0)	< 0.001 (0.87)
13-HOTE	0.004 (0.58)	0.002 (0.72)	0.008 (0.43)	0.009 (0.40)
12-HEPE	0.013 (0.32)	0.008 (0.44)	0.032 (0.11)	0.019 (0.22)
5-HETE	0.011 (0.35)	0.023 (0.18)	0.028 (0.14)	0.011 (0.35)
12-HETE	0.220 (0.19)	0.007 (0.46)	0.002 (0.72)	0.007 (0.47)
12-HpETE	0.016 (0.26)	0.005 (0.54)	0.033 (0.11)	0.024 (0.17)
**Antioxidant**				
**enzymes**	0.004 (0.57)	0.030 (0.11)	0.02 (0.20)	0.025 (0.15)
SOD	**0.051 (0.038)**	0.013 (0.30)	0.016 (0.25)	0.013 (0.29)
GPX	0.003 (0.59)	0.012 (0.32)	0.012 (0.33)	0.009 (0.38)
GSHR	0.013 (0.29)	0.006 (0.47)	0.03 (0.11)	0.011 (0.33)
GST	0.011 (0.34)	< 0.001 (0.94)	0.010 (0.35)	0.009 (0.38)
PON-1-ARYL	0.019 (0.21)	0.011 (0.34)	0.002 (0.67)	0.013 (0.30)
PON-1 PXON				

Abbreviations: BHB: Beta-Hydroxybutyrate; HDL-C: High density lipoprotein cholesterol, LDL-C: Low density lipoprotein cholesterol; Apo: Apolipoprotein; HO: Hydroxy; Hp: hydroperoxy; ODE: octadecadienoic acid; OTE: octadecatrienoic acid; ETE: eicosatetraenoic acid; HODE: Hydroxy octadecadienoic acid; HOTE: Hydroxy octadecatrienoic fatty acid; HETE: Hydroxy eicosatetraenoic acid; SOD: Superoxide dismutase; GPX: Glutathione

peroxidase; GSHR: Glutathione reductase; GST: Glutathione-S-transferase; ARYL: PON-1 arylesterase activity, PON-1: Paraoxonase-1; PXON- Paraoxonase activity of PON-1.
